# From Detection to Delay: Real-World Gaps in Post-Cologuard^®^ Colonoscopy Adherence

**DOI:** 10.1007/s12029-025-01350-5

**Published:** 2025-11-18

**Authors:** Muhammad Ali Ibrahim Kazi, Imran Qureshi, Akshay Sharma, Nirav Agrawal, Mahnoor Sarfraz, Barry Meisenberg, Sanmeet Singh

**Affiliations:** 1 Anne Arundel Medical Center, Annapolis, MD USA; 2 Rutgers New Jersey Medical School, Newark, NJ USA; 3 Aga Khan University, Karachi, Pakistan

**Keywords:** Colorectal cancer, Cancer screening, Cologuard, Colonoscopy adherence, Care navigation

## Abstract

**Purpose:**

Colorectal cancer remains a leading cause of cancer deaths, highlighting the need for early detection. Cologuard^®^, a non-invasive stool DNA test, detects biomarkers for CRC and precancerous lesions but requires follow-up colonoscopy and has a high false-positive rate. This study evaluates colonoscopy follow-up rates and diagnostic outcomes after positive Cologuard^®^ results.

**Methods:**

We conducted a retrospective cohort study using the TriNetX database, a global federated real-world data platform, to analyze patients aged ≥ 18 years who tested positive on Cologuard. The primary outcome was whether patients underwent an endoscopic procedure (colonoscopy) within 12 months of a positive result. The secondary outcome was the diagnoses made during follow-up colonoscopy, including malignant neoplasms (colorectal cancer) and benign neoplasms (polyps).

**Results:**

A total of 3,916 patients underwent Cologuard^®^ testing, with 61.3% being female, 35% male, and 3.7% other genders. Of the 385 patients who tested positive for Cologuard^®^ (mean age 65 ± 8.75 years), 171 (44%) underwent follow-up colonoscopy within 12 months. Of these, 10 cases (5.8%) were diagnosed with malignant neoplasms, and 56 cases (32.7%) were diagnosed with benign neoplasms (polyps).

**Conclusion:**

The study found poor follow-up adherence, with only 44% completing colonoscopy and a high false positive rate with just 38.5% of positive Cologuard^®^ results showing significant lesions. These findings emphasize the need for better patient education, streamlined care pathways, and improved communication to enhance follow-up compliance.

## Introduction

Cologuard^®^ is a noninvasive, at-home screening test which is prescribed to detect colorectal cancer and advanced precancerous lesions in patients who are aged 45 and older and are at risk [1]. The test functions by identifying abnormal DNA markers found to be associated with colorectal neoplasia as well as the presence of occult hemoglobin in the stool, which both serve as potential indicators of cancerous or precancerous lesions in the colon [2]. It is a test that has risen in popularity. The use of DNA stool tests for colorectal cancer screening has increased more than nine-fold since 2018, rising from 3% of screenings in the first quarter of 2018 to 31% of screenings in the fourth quarter of 2023 [3].

The Cologuard^®^ has exhibited a high sensitivity for detecting CRC in clinical studies making it an important tool for screening and early detection. The Cologuard^®^ test has two generations having undergone improvement in sensitivity and specificity. The first generation demonstrated a sensitivity of 92% for colorectal cancer and 42% for precancerous lesions [1]. One study showed that the Cologuard^®^ detected significantly more cancers than other stool-based tests but also gave more false positive tests [1]. In comparison FIT test had a sensitivity of 73.8% for colorectal cancer and 23.8% for pre-cancerous lesions. This shows that Cologuard has increased sensitivity but decreased specificity yielding more false positives requiring colonoscopy. Apart from that FIT is recommended yearly while the Cologuard test is recommended after 3 years. Studies have shown that adherence to yearly testing remains poor with one study having repeat testing of 23.4% in year 2 and 10.6% in year 3 [4].

However, as a stool-based test it presents with limitations as it does not provide direct visualization of the colon and cannot be used to identify or remove polyps and unable to identify other colorectal diseases [5]. Despite this drawback, the Cologuard^®^ is widely used as a convenient and effective screening option, particularly for individuals who may be reluctant to undergo a colonoscopy [3]. While the Cologuard^®^ is highly sensitive in detecting colorectal cancer or precancerous lesions, it remains a screening tool rather than a diagnostic test [3]. Suboptimal rates of follow-up represent a missed opportunity in early detection and prevention.

Timely follow-up is essential, as delays in colonoscopy following a positive stool-based test have been associated with an increased risk of colorectal cancer progression and mortality [6]. Barriers to timely colonoscopy include lack of awareness, psychological barriers, limited access to specialists, and financial constraints, all of which can negatively impact patient outcomes [7]. By implementing timely diagnostic follow-up, screening programs can maximize the benefits of early detection and intervention, ultimately reducing the burden of colorectal cancer [8].

However, despite well documented performance metrics, a significant research gap remains in understanding how many patients with positive Cologuard^®^ tests follow up with the recommended colonoscopy. The objective of this study is to assess whether patients who receive a positive Cologuard^®^ test undergo a timely follow-up colonoscopy, which is essential for confirming the presence of colorectal cancer or precancerous lesions. By analyzing patient data, and potential barriers to timely follow-up, this study aims to identify gaps in adherence and areas for improvement.

## Methods

We conducted a retrospective cohort study using the TriNetX database, a global federated real-world data platform [9]. We used it to analyze the follow-up patterns of patients who tested positive on Cologuard^®^, a multi-target stool DNA test for colorectal cancer screening. The study included adult patients aged ≥ 18 years who underwent Cologuard^®^ testing, identified using relevant diagnostic and procedural codes within the database. Data was retrospective and collected from 2016 to 2024.

The primary outcome was the proportion of patients who underwent an endoscopic procedure (colonoscopy) within 12 months following a positive Cologuard^®^ result. The secondary outcomes included the diagnostic findings from follow-up colonoscopy, specifically the presence of malignant neoplasms (colorectal cancer) and benign neoplasms (polyps). Additional variables assessed included patient demographics such as age, sex, and other gender identities. Descriptive statistics were used to summarize baseline characteristics, and outcome proportions were reported as percentages.

## Results

A total of 3,916 patients underwent Cologuard^®^ testing. Among these, 61.3% were female, 35% were male, and 3.7% identified as other genders. The total cohort, 385 patients (mean age 65 ± 8.75 years) had a positive Cologuard^®^ result and were included in the final analysis.

Among the patients who tested positive, 171 (44%) underwent follow-up colonoscopy within 12 months. Among those who underwent colonoscopy, 10 patients (5.8%) were diagnosed with malignant neoplasms (colorectal cancer), and 56 patients (32.7%) were diagnosed with benign neoplasms (polyps). The remaining patients who underwent colonoscopy did not have significant neoplastic findings.

## Discussion

Our study highlights an important gap in patient’s adherence to follow-up following a positive Cologuard^®^ test, with 44% of patients completing a colonoscopy in one year. This low percentage shows important barriers to timely completion such as healthcare access, patient perceptions and logistical challenges [10]. These findings are aligned with previous research which indicates that noninvasive screening tests, like the Cologuard^®^ test are effective in increasing overall screening rates but there might be suboptimal adherence in turn delaying diagnosis of CRC or precancerous lesions [11].

Colonoscopy plays a crucial role in (CRC) screening particularly in the context of a positive Cologuard^®^ test as it remains the gold standard for detecting and removing precancerous lesions and early-stage malignancies. Unlike stool-based tests, which primarily indicate the presence of abnormal DNA or blood, colonoscopy enables the identification and removal of adenomatous polyps before they progress to cancer [12]. In the case of a positive Cologuard^®^ test, timely follow-up colonoscopy is essential to confirm or rule out malignancy. Failure to complete a follow-up colonoscopy may lead to missed opportunities for early detection and treatment, potentially resulting in more advanced disease at diagnosis and worse clinical outcomes [6].

Addressing the barriers to colonoscopy adherence is therefore crucial to optimizing the effectiveness of CRC screening programs. Several strategies to increase adherence can be implemented including targeted patient education on the importance of confirmatory colonoscopy [13]. Patient navigation programs can help patients in overcoming logistical obstacles such as scheduling issues and insurance limitations [14].

**Fig. 1:**
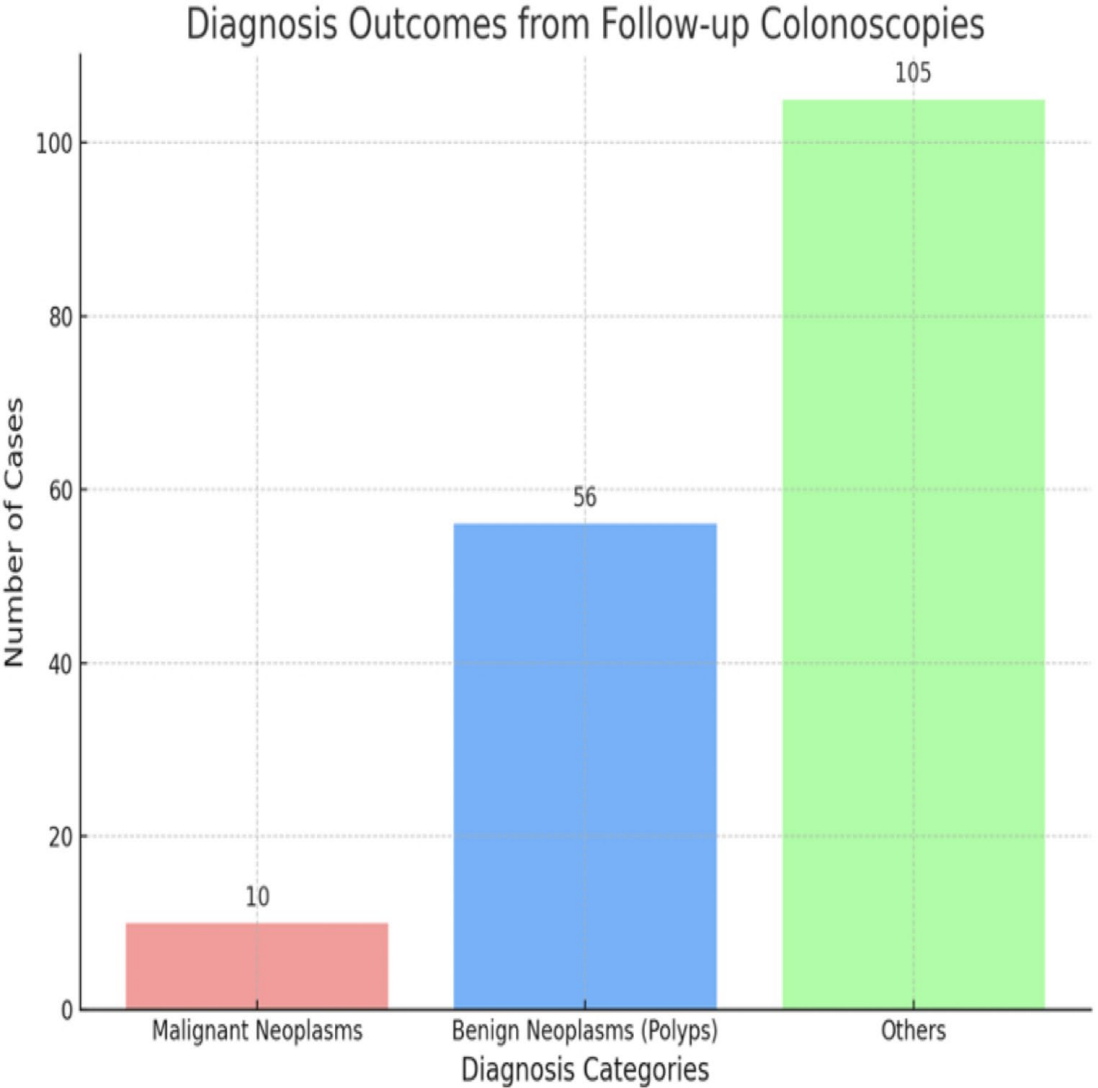
Bar chart showing the distribution of findings among patients who underwent follow-up colonoscopy after positive stool testing

Another important highlight of the study showed the diagnostic yield of follow-up colonoscopies which revealed that a minority of patients with positive Cologuard^®^ results were diagnosed with CRC (5.8%), while a larger proportion (32.7%) had benign polyps as shown in Fig. 1. This reinforces the utility of Cologuard^®^ in detecting precancerous lesions but also raises concerns regarding its specificity. Notably, 61.5% of patients had no significant findings on colonoscopy, suggesting a considerable rate of false positives. While this limitation is inherent to many screening modalities and the specificity of the test has been improved with the second generation of Cologuard^®^ test, excessive false positives may contribute negatively to healthcare costs [15] This leads to increased patient anxiety, unnecessary procedures and reduces willingness to have future screenings [16]. Future research should explore refining screening algorithms to improve specificity and reduce unnecessary colonoscopies while maintaining high sensitivity for CRC detection.

In conclusion, while Cologuard^®^ plays an important role in CRC screening, improving adherence to follow-up colonoscopy is essential to maximizing its clinical utility. Efforts to address healthcare disparities, streamline follow-up processes, and refine risk stratification methods will be key to enhancing the overall effectiveness of CRC screening programs and improving patient outcomes.

## Data Availability

The data that support the findings of this study are available from the TriNetX platform, but restrictions apply to the availability of these data, which were used under license for the current study and are not publicly available. Data may be available from the corresponding author upon reasonable request and with permission from TriNetX.
